# *N*,*N*′-Dicarb­oxy-*N*,*N*′-dicarboxyl­ato(*m*-phenyl­ene)dimethanaminium monohydrate

**DOI:** 10.1107/S1600536811013675

**Published:** 2011-04-22

**Authors:** Yu-Xing Qiang, Shou-Rong Zhu, Min Shao

**Affiliations:** a Department of Chemistry, Shanghai University, Shanghai 200444, People’s Republic of China; b Instrumental Analysis Center, Shanghai University, Shanghai 200444, People’s Republic of China

## Abstract

In the title inner salt, C_16_H_20_N_2_O_8_·H_2_O, two of four carboxyl groups are deprotonated, while the two imine groups are protonated. The two imino­diacetate groups are located on the same side of the benzene ring plane. Extensive inter­molecular O—H⋯O and N—H⋯O hydrogen bonds occur in the crystal.

## Related literature

The title compound tends to form dinuclear metal complexes, which are capable of di­oxy­gen activation, see: Furutachi *et al.* (2003 ▶); Zhao *et al.* (2008*a* ▶,*b* ▶). For the structures of aromatic-substituted imino­diacetic acids, see: Choquesillo-Laza­rte *et al.* (2002 ▶); Sánchez-Moreno *et al.* (2003 ▶).
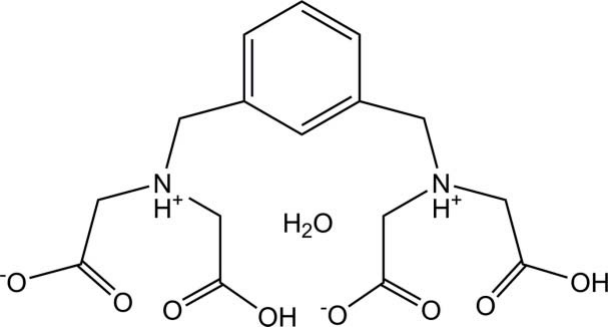


## Experimental

### 

#### Crystal data

C_16_H_20_N_2_O_8_·H_2_O
*M* *_r_* = 386.36
Monoclinic, 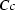

*a* = 22.491 (3) Å
*b* = 5.4342 (7) Å
*c* = 14.3118 (19) Å
β = 106.788 (2)°
*V* = 1674.6 (4) Å^3^
*Z* = 4
Mo *K*α radiation
μ = 0.13 mm^−1^
*T* = 296 K
0.20 × 0.10 × 0.10 mm

#### Data collection

Bruker APEXII CCD diffractometer
Absorption correction: multi-scan (*SADABS*; Sheldrick, 2004 ▶) *T*_min_ = 0.975, *T*_max_ = 0.988
4927 measured reflections
1891 independent reflections
1701 reflections with *I* > 2σ(*I*)
*R*_int_ = 0.020

#### Refinement

*R*[*F* ^2^ > 2σ(*F* ^2^)] = 0.030
*wR*(*F* ^2^) = 0.069
*S* = 0.97
1891 reflections
252 parameters
2 restraints
H atoms treated by a mixture of independent and constrained refinement
Δρ_max_ = 0.13 e Å^−3^
Δρ_min_ = −0.15 e Å^−3^

### 

Data collection: *APEX2* (Bruker, 2001 ▶); cell refinement: *SAINT* (Bruker, 2001 ▶); data reduction: *SAINT*; program(s) used to solve structure: *SHELXTL* (Sheldrick, 2008 ▶); program(s) used to refine structure: *SHELXTL*; molecular graphics: *SHELXTL*; software used to prepare material for publication: *SHELXTL*.

## Supplementary Material

Crystal structure: contains datablocks I, global. DOI: 10.1107/S1600536811013675/xu5178sup1.cif

Structure factors: contains datablocks I. DOI: 10.1107/S1600536811013675/xu5178Isup2.hkl

Additional supplementary materials:  crystallographic information; 3D view; checkCIF report

## Figures and Tables

**Table 1:** Hydrogen-bond geometry (Å, °)

*D*—H⋯*A*	*D*—H	H⋯*A*	*D*⋯*A*	*D*—H⋯*A*
N1—H1*A*⋯O8^i^	0.96 (3)	2.46 (3)	3.216 (3)	135 (2)
N2—H2*A*⋯O6^ii^	0.97 (3)	2.03 (3)	2.896 (3)	148 (2)
O1*W*—H1*WB*⋯O8^ii^	0.85	2.02	2.847 (3)	165
O1*W*—H1*WA*⋯O7	0.85	1.88	2.729 (3)	174
O4—H4*A*⋯O1*W*^iii^	0.85	1.71	2.552 (3)	171
O5—H5*A*⋯O1^iv^	0.85	1.65	2.472 (3)	161

Symmetry codes: (i) 
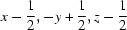
; (ii) 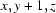; (iii) 
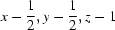
; (iv) 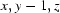.

**Fig. 1.**
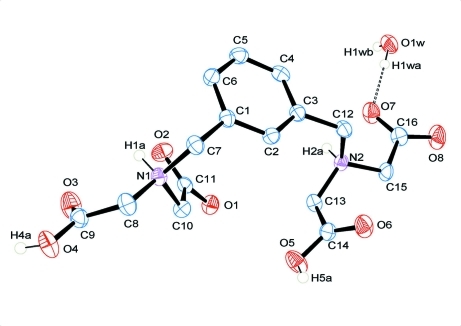
The molecular structure of the title compound with displacement ellipsoids drawn at the 50% probability level.

**Fig. 2.**
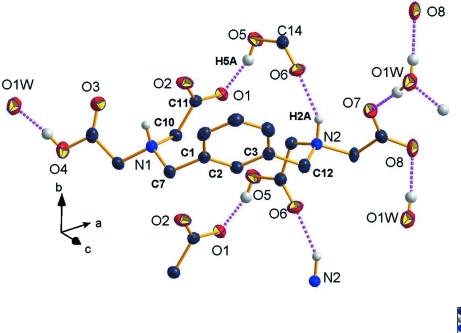
H-bonds in the title compound. Purple bonds are H-Bond.

**Fig. 3.**
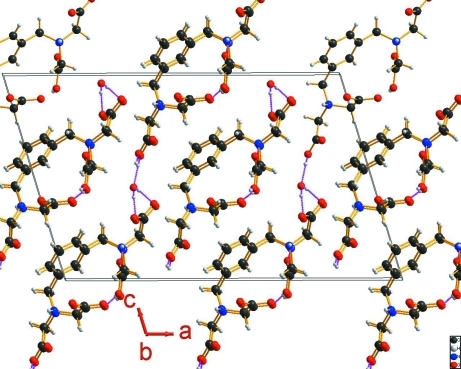
Crystal packing diagram of the title compound.

## supplementary crystallographic information

### Comment

The title compound tends to form dinuclear complexes with transition metal ions. The dinuclear complexes are capable for dioxygen activation (Furutachi *et al.*, 2003; Zhao *et al.*, 2008*a*). Structures of their dinuclear complexes have been reported. As a part of serial structural investigation of dioxygen activation by dinuclear complexes, the title compound was prepared in the laboratory and its X-ray structure is presented here.

The molecular structure of the title compound is shown in Fig.1. The compound is not symmetry as the scheme. The asymmetric moiety contains a whole molecule. There are two protons bind to two carboxylate group (O4 and O5). Two protons bind to the imino nitrogen atom N1 and N2 with N—H distances of 0.960 (27) and 0.968 (27)Å respectively, which is 0.1 Å longer than O—H distances (0.851 (2) Å. Different from its zinc(II) complex, the two iminodiacetic moiety are in the opposite side with respect to the central benzyl ring (Zhao *et al.*, 2008*a*). The phenyl group, [C1/C2/C3/C4/C5/C6]and methylene C7 and C12 are in the same plane, but the N1/C7/C1 and N2/C12/C3 planes are almost perpendicular to the phenyl plane with dihedral angle of 89.4 (3) and 88.6 (3)° respectively, which is comparable to those in *N*-(*p*-nitrobenzyl)iminodiacetic acid (Sánchez-Moreno *et al.*, 2003), but quite different from the almost coplanar geometry in *N*-(2-pyridylmethyl)iminodiacetic acid (Choquesillo-Lazarte *et al.*, 2002). N1/C7/C1 and N2/C12/C3 planes have a dihydral angle of 59.7 (1)°. The bond lengths and angles are in normal ranges and comparable to the above mentioned compounds and its complex (Zhao *et al.*, 2008*a*; Zhao *et al.*, 2008*b*). In the compound, the two carboxylate for both iminoaiacetic group are far away with C(9)—C(11) and C(14)—C(16) diantances at 4.089 (3) and 4.667 (3)Å respectively. There are intermolecular H-bonds in the compound. No intramolecular H-bond found in the compound(Fig.2).This is quite different from *N*-(*p*-nitrobenzyl) iminodiacetic acid (Sánchez-Moreno *et al.*, 2003) and *N*-(2-pyridylmethyl)iminodiacetic acid (Choquesillo-Lazarte *et al.*, 2002). In these literatures, there is a proton binding to the imino nitrogen atom. There are N—H···O intramolecular H-bonds. In the title compound, carboxyalte O6/C14/O5 from other molecule links the molecules *via* O1 and N2 through H—bond to form a pseudo 15–membered ring [H5a/O5/C14/O6/H2a/N2/C12/C3/C2/C1/C7/N1/C10/C11/O1] (Fig.2). O5—H5A···O1ii has the shortest distance of 2.472 (3)Å in all H—bonds. N—H···O H—bond is much weaker than corresponding O—H···O H—bond. It is the intermolecular H-bonds that bind adjacent molecules as shown in Fig. 3 and table 2.

### Experimental

To a mixture of bromoacetic acid (41.816 g, 0.30 mol) and lithium hydroxide monohydrate (12.627 g, 0.30 mol) in water (100 ml) containing phenolphthalein was added *m*-xylenediamine (10.0 g, 73.4 mmol). The reaction mixture was stirred at 70°C for 3 h and during the reaction, pH was maintained at 10 by addition of lithium hydroxide monohydrate (12.627 g, 0.30 mol). After the mixture cooled to ambient temperature, the solution was made acidic (pH = 1) by addition of conc. HCl to give white powder. Yield: 24.3 g (84%).

0.0194 g (0.05 mmol) of the white powder was added 0.5 ml 0.1 mol/*L* KOH solution, then 5 ml sub-boiled water was added to give a clear solution. Gradually add 0.1 mol/*L* HNO_3_, to adjust the pH of the solution to 5. The solution was allowed to stand at room temperature for 3 days. Colorless block crystals suitable for crystal diffraction were obtained.

### Refinement

H atoms bonded to N atoms were located in a difference map and refined isotropically. Other H atoms were positioned geometrically and refined using a riding model with C—H = 0.93–0.97 Å and O—H = 0.85 Å; *U*_iso_(H) = 1.2*U*_eq_(C,*O*). As no significant anomalous scattering, Friedels pairs were merged.

### Crystal data

**Table:**

C_16_H_20_N_2_O_8_·H_2_O	*F*(000) = 816
*M**_r_* = 386.36	*D*_x_ = 1.532 Mg m^−^^3^
Monoclinic, *C**c*	Mo *K*α radiation, λ = 0.71073 Å
Hall symbol: C -2yc	Cell parameters from 1912 reflections
*a* = 22.491 (3) Å	θ = 3.0–27.3°
*b* = 5.4342 (7) Å	µ = 0.13 mm^−^^1^
*c* = 14.3118 (19) Å	*T* = 296 K
β = 106.788 (2)°	Block, colorless
*V* = 1674.6 (4) Å^3^	0.20 × 0.10 × 0.10 mm
*Z* = 4	

### Data collection

**Table:**

Bruker APEXII CCD diffractometer	1891 independent reflections
Radiation source: fine-focus sealed tube	1701 reflections with *I* > 2σ(*I*)
graphite	*R*_int_ = 0.020
φ and ω scans	θ_max_ = 27.5°, θ_min_ = 1.9°
Absorption correction: multi-scan (*SADABS*; Sheldrick, 2004)	*h* = −28→18
*T*_min_ = 0.975, *T*_max_ = 0.988	*k* = −6→7
4927 measured reflections	*l* = −18→18

### Refinement

**Table:**

Refinement on *F*^2^	Primary atom site location: structure-invariant direct methods
Least-squares matrix: full	Secondary atom site location: difference Fourier map
*R*[*F*^2^ > 2σ(*F*^2^)] = 0.030	Hydrogen site location: inferred from neighbouring sites
*wR*(*F*^2^) = 0.069	H atoms treated by a mixture of independent and constrained refinement
*S* = 0.97	*w* = 1/[σ^2^(*F*_o_^2^) + (0.0302*P*)^2^ + 1.0337*P*] where *P* = (*F*_o_^2^ + 2*F*_c_^2^)/3
1891 reflections	(Δ/σ)_max_ < 0.001
252 parameters	Δρ_max_ = 0.13 e Å^−^^3^
2 restraints	Δρ_min_ = −0.15 e Å^−^^3^

### Special details

**Table:**

Experimental. Anal. Calcd for C_16_H_22_N_2_O_9_: C, 49.73; H, 5.64; N, 7.34%. Found: C, 49.69; H, 5.64; N, 7.34%. ^1^H NMR (D_2_O in the presence of K_2_CO_3_): *d* (p.p.m.) = 3.31 (8*H*, s, NCH_2_CO_2_), 3.95 (4*H*, s, PhCH_2_N), 7.36 – 7.41 (4*H*, m, PhH).
Geometry. All e.s.d.'s (except the e.s.d. in the dihedral angle between two l.s. planes) are estimated using the full covariance matrix. The cell e.s.d.'s are taken into account individually in the estimation of e.s.d.'s in distances, angles and torsion angles; correlations between e.s.d.'s in cell parameters are only used when they are defined by crystal symmetry. An approximate (isotropic) treatment of cell e.s.d.'s is used for estimating e.s.d.'s involving l.s. planes.
Refinement. Refinement of *F*^2^ against ALL reflections. The weighted *R*-factor *wR* and goodness of fit *S* are based on *F*^2^, conventional *R*-factors *R* are based on *F*, with *F* set to zero for negative *F*^2^. The threshold expression of *F*^2^ > σ(*F*^2^) is used only for calculating *R*-factors(gt) *etc*. and is not relevant to the choice of reflections for refinement. *R*-factors based on *F*^2^ are statistically about twice as large as those based on *F*, and *R*- factors based on ALL data will be even larger.

### Fractional atomic coordinates and isotropic or equivalent isotropic displacement parameters (Å^2^)

**Table:**

	*x*	*y*	*z*	*U*_iso_*/*U*_eq_	
C1	0.48342 (11)	0.0785 (4)	0.53137 (16)	0.0231 (5)	
C2	0.54217 (11)	−0.0180 (4)	0.57446 (17)	0.0240 (5)	
H2	0.5537	−0.1655	0.5516	0.029*	
C3	0.58405 (11)	0.1035 (5)	0.65160 (17)	0.0243 (5)	
C4	0.56643 (12)	0.3244 (5)	0.68491 (17)	0.0281 (5)	
H4	0.5944	0.4095	0.7352	0.034*	
C5	0.50736 (13)	0.4184 (5)	0.64350 (18)	0.0296 (5)	
H5	0.4955	0.5642	0.6671	0.036*	
C6	0.46596 (12)	0.2960 (5)	0.56708 (18)	0.0272 (5)	
H6	0.4264	0.3596	0.5396	0.033*	
C7	0.43870 (11)	−0.0521 (5)	0.44711 (17)	0.0246 (5)	
H7A	0.3971	−0.0390	0.4538	0.030*	
H7B	0.4495	−0.2253	0.4495	0.030*	
C8	0.39405 (12)	−0.0909 (5)	0.27035 (18)	0.0285 (5)	
H8A	0.3588	−0.1388	0.2925	0.034*	
H8B	0.4139	−0.2395	0.2567	0.034*	
C9	0.37171 (11)	0.0596 (5)	0.17845 (18)	0.0286 (6)	
C10	0.50246 (11)	0.0599 (4)	0.33451 (18)	0.0245 (5)	
H10A	0.4991	0.0363	0.2660	0.029*	
H10B	0.5276	−0.0724	0.3711	0.029*	
C11	0.53354 (11)	0.3057 (4)	0.36863 (17)	0.0233 (5)	
C12	0.64659 (11)	−0.0044 (5)	0.70305 (17)	0.0264 (5)	
H12A	0.6431	−0.1823	0.7033	0.032*	
H12B	0.6590	0.0508	0.7704	0.032*	
C13	0.68289 (12)	−0.0009 (4)	0.55037 (17)	0.0257 (5)	
H13A	0.7183	0.0447	0.5282	0.031*	
H13B	0.6477	0.0960	0.5134	0.031*	
C14	0.66863 (11)	−0.2693 (5)	0.52703 (18)	0.0258 (5)	
C15	0.75870 (11)	−0.0232 (5)	0.71783 (18)	0.0283 (6)	
H15A	0.7870	−0.0372	0.6781	0.034*	
H15B	0.7541	−0.1851	0.7435	0.034*	
C16	0.78576 (12)	0.1544 (5)	0.80242 (18)	0.0293 (5)	
H1A	0.4248 (13)	0.219 (5)	0.347 (2)	0.028 (7)*	
H2A	0.6999 (12)	0.244 (5)	0.658 (2)	0.029 (7)*	
N1	0.43897 (9)	0.0518 (4)	0.34879 (14)	0.0228 (4)	
N2	0.69672 (9)	0.0663 (4)	0.65600 (14)	0.0225 (4)	
O1	0.59162 (8)	0.3058 (3)	0.37755 (12)	0.0295 (4)	
O2	0.50164 (9)	0.4792 (3)	0.38026 (14)	0.0342 (4)	
O3	0.38729 (10)	0.2699 (4)	0.17322 (15)	0.0441 (5)	
O4	0.33302 (9)	−0.0662 (4)	0.10907 (14)	0.0411 (5)	
H4A	0.3175	0.0277	0.0606	0.049*	
O5	0.64514 (10)	−0.3008 (4)	0.43420 (13)	0.0401 (5)	
H5A	0.6336	−0.4497	0.4230	0.048*	
O6	0.67714 (10)	−0.4290 (3)	0.58908 (14)	0.0391 (5)	
O7	0.76142 (10)	0.3614 (4)	0.79623 (14)	0.0398 (5)	
O8	0.83165 (10)	0.0772 (4)	0.86778 (15)	0.0472 (6)	
O1W	0.78493 (9)	0.6735 (4)	0.95209 (13)	0.0399 (5)	
H1WA	0.7763	0.5697	0.9057	0.048*	
H1WB	0.7988	0.8079	0.9363	0.048*	

### Atomic displacement parameters (Å^2^)

**Table:**

	*U*^11^	*U*^22^	*U*^33^	*U*^12^	*U*^13^	*U*^23^
C1	0.0222 (11)	0.0252 (12)	0.0219 (11)	−0.0016 (9)	0.0064 (9)	0.0030 (10)
C2	0.0241 (12)	0.0230 (12)	0.0252 (11)	0.0007 (10)	0.0076 (9)	−0.0015 (10)
C3	0.0229 (12)	0.0274 (12)	0.0223 (11)	−0.0007 (10)	0.0062 (9)	0.0013 (10)
C4	0.0319 (14)	0.0317 (14)	0.0206 (12)	−0.0044 (11)	0.0076 (10)	−0.0047 (10)
C5	0.0388 (14)	0.0238 (12)	0.0286 (13)	0.0033 (11)	0.0133 (11)	−0.0013 (10)
C6	0.0256 (12)	0.0297 (13)	0.0270 (13)	0.0054 (10)	0.0088 (10)	0.0041 (10)
C7	0.0213 (12)	0.0288 (13)	0.0228 (12)	−0.0038 (9)	0.0048 (9)	0.0009 (10)
C8	0.0267 (12)	0.0286 (14)	0.0267 (12)	−0.0083 (10)	0.0023 (10)	0.0008 (10)
C9	0.0224 (13)	0.0371 (15)	0.0253 (13)	−0.0012 (10)	0.0053 (10)	−0.0003 (10)
C10	0.0228 (12)	0.0233 (12)	0.0274 (12)	−0.0014 (10)	0.0074 (9)	−0.0005 (10)
C11	0.0282 (13)	0.0220 (11)	0.0186 (10)	−0.0014 (10)	0.0051 (9)	0.0018 (9)
C12	0.0247 (13)	0.0321 (13)	0.0210 (11)	−0.0007 (10)	0.0047 (10)	0.0010 (10)
C13	0.0272 (12)	0.0253 (12)	0.0225 (12)	−0.0023 (10)	0.0039 (9)	0.0020 (9)
C14	0.0232 (12)	0.0264 (12)	0.0268 (12)	0.0003 (10)	0.0056 (10)	0.0008 (10)
C15	0.0204 (12)	0.0309 (14)	0.0273 (13)	0.0025 (10)	−0.0032 (10)	−0.0017 (10)
C16	0.0266 (13)	0.0293 (13)	0.0287 (13)	−0.0069 (10)	0.0027 (10)	−0.0019 (11)
N1	0.0214 (10)	0.0227 (10)	0.0221 (10)	−0.0017 (8)	0.0031 (8)	0.0006 (8)
N2	0.0209 (10)	0.0196 (10)	0.0237 (10)	−0.0005 (8)	0.0012 (8)	−0.0008 (8)
O1	0.0246 (9)	0.0267 (9)	0.0342 (10)	−0.0039 (7)	0.0035 (7)	−0.0010 (8)
O2	0.0398 (11)	0.0212 (9)	0.0443 (11)	0.0007 (8)	0.0161 (9)	−0.0021 (8)
O3	0.0479 (12)	0.0371 (12)	0.0392 (12)	−0.0087 (10)	−0.0007 (9)	0.0086 (9)
O4	0.0414 (12)	0.0467 (12)	0.0265 (9)	−0.0075 (9)	−0.0039 (8)	0.0024 (9)
O5	0.0556 (13)	0.0334 (11)	0.0257 (9)	−0.0153 (9)	0.0030 (9)	−0.0036 (8)
O6	0.0559 (13)	0.0224 (9)	0.0333 (10)	0.0002 (9)	0.0037 (9)	0.0041 (8)
O7	0.0476 (12)	0.0297 (11)	0.0325 (10)	−0.0013 (9)	−0.0037 (8)	−0.0042 (8)
O8	0.0378 (11)	0.0477 (13)	0.0397 (11)	0.0021 (10)	−0.0149 (9)	−0.0032 (10)
O1W	0.0443 (12)	0.0452 (12)	0.0258 (9)	0.0013 (9)	0.0031 (8)	−0.0015 (9)

### Geometric parameters (Å, °)

**Table:**

C1—C6	1.388 (3)	C10—H10B	0.9700
C1—C2	1.389 (3)	C11—O2	1.225 (3)
C1—C7	1.507 (3)	C11—O1	1.275 (3)
C2—C3	1.394 (3)	C12—N2	1.520 (3)
C2—H2	0.9300	C12—H12A	0.9700
C3—C4	1.391 (4)	C12—H12B	0.9700
C3—C12	1.505 (3)	C13—N2	1.498 (3)
C4—C5	1.387 (4)	C13—C14	1.510 (3)
C4—H4	0.9300	C13—H13A	0.9700
C5—C6	1.385 (4)	C13—H13B	0.9700
C5—H5	0.9300	C14—O6	1.216 (3)
C6—H6	0.9300	C14—O5	1.292 (3)
C7—N1	1.518 (3)	C15—N2	1.500 (3)
C7—H7A	0.9700	C15—C16	1.530 (3)
C7—H7B	0.9700	C15—H15A	0.9700
C8—N1	1.492 (3)	C15—H15B	0.9700
C8—C9	1.506 (4)	C16—O7	1.243 (3)
C8—H8A	0.9700	C16—O8	1.248 (3)
C8—H8B	0.9700	N1—H1A	0.96 (3)
C9—O3	1.204 (3)	N2—H2A	0.97 (3)
C9—O4	1.308 (3)	O4—H4A	0.8501
C10—N1	1.501 (3)	O5—H5A	0.8500
C10—C11	1.521 (3)	O1W—H1WA	0.8500
C10—H10A	0.9700	O1W—H1WB	0.8500
			
C6—C1—C2	119.5 (2)	O1—C11—C10	113.3 (2)
C6—C1—C7	120.1 (2)	C3—C12—N2	113.12 (19)
C2—C1—C7	120.4 (2)	C3—C12—H12A	109.0
C1—C2—C3	120.7 (2)	N2—C12—H12A	109.0
C1—C2—H2	119.6	C3—C12—H12B	109.0
C3—C2—H2	119.6	N2—C12—H12B	109.0
C4—C3—C2	119.1 (2)	H12A—C12—H12B	107.8
C4—C3—C12	119.1 (2)	N2—C13—C14	115.3 (2)
C2—C3—C12	121.7 (2)	N2—C13—H13A	108.4
C3—C4—C5	120.3 (2)	C14—C13—H13A	108.4
C3—C4—H4	119.9	N2—C13—H13B	108.4
C5—C4—H4	119.9	C14—C13—H13B	108.4
C6—C5—C4	120.2 (2)	H13A—C13—H13B	107.5
C6—C5—H5	119.9	O6—C14—O5	126.0 (2)
C4—C5—H5	119.9	O6—C14—C13	123.3 (2)
C5—C6—C1	120.2 (2)	O5—C14—C13	110.7 (2)
C5—C6—H6	119.9	N2—C15—C16	110.6 (2)
C1—C6—H6	119.9	N2—C15—H15A	109.5
C1—C7—N1	112.71 (19)	C16—C15—H15A	109.5
C1—C7—H7A	109.0	N2—C15—H15B	109.5
N1—C7—H7A	109.0	C16—C15—H15B	109.5
C1—C7—H7B	109.0	H15A—C15—H15B	108.1
N1—C7—H7B	109.0	O7—C16—O8	127.5 (2)
H7A—C7—H7B	107.8	O7—C16—C15	116.6 (2)
N1—C8—C9	111.0 (2)	O8—C16—C15	115.8 (2)
N1—C8—H8A	109.4	C8—N1—C10	112.15 (19)
C9—C8—H8A	109.4	C8—N1—C7	108.73 (18)
N1—C8—H8B	109.4	C10—N1—C7	113.33 (18)
C9—C8—H8B	109.4	C8—N1—H1A	109.1 (17)
H8A—C8—H8B	108.0	C10—N1—H1A	106.9 (16)
O3—C9—O4	126.2 (3)	C7—N1—H1A	106.4 (17)
O3—C9—C8	122.7 (2)	C15—N2—C13	113.76 (19)
O4—C9—C8	111.1 (2)	C15—N2—C12	109.77 (19)
N1—C10—C11	110.46 (19)	C13—N2—C12	114.90 (19)
N1—C10—H10A	109.6	C15—N2—H2A	104.9 (16)
C11—C10—H10A	109.6	C13—N2—H2A	105.7 (16)
N1—C10—H10B	109.6	C12—N2—H2A	107.0 (17)
C11—C10—H10B	109.6	C9—O4—H4A	109.4
H10A—C10—H10B	108.1	C14—O5—H5A	109.4
O2—C11—O1	127.5 (2)	H1WA—O1W—H1WB	112.4
O2—C11—C10	119.1 (2)		

### Hydrogen-bond geometry (Å, °)

**Table:**

*D*—H···*A*	*D*—H	H···*A*	*D*···*A*	*D*—H···*A*
N1—H1A···O8^i^	0.96 (3)	2.46 (3)	3.216 (3)	135 (2)
N2—H2A···O6^ii^	0.97 (3)	2.03 (3)	2.896 (3)	148 (2)
O1W—H1WB···O8^ii^	0.85	2.02	2.847 (3)	165
O1W—H1WA···O7	0.85	1.88	2.729 (3)	174
O4—H4A···O1W^iii^	0.85	1.71	2.552 (3)	171
O5—H5A···O1^iv^	0.85	1.65	2.472 (3)	161

Symmetry codes: (i) *x*−1/2, −*y*+1/2, *z*−1/2; (ii) *x*, *y*+1, *z*; (iii) *x*−1/2, *y*−1/2, *z*−1; (iv) *x*, *y*−1, *z*.
